# A Hazelnut-Enriched Diet Modulates Oxidative Stress and Inflammation Gene Expression without Weight Gain

**DOI:** 10.1155/2019/4683723

**Published:** 2019-07-04

**Authors:** Laura Di Renzo, Giorgia Cioccoloni, Sergio Bernardini, Ludovico Abenavoli, Vincenzo Aiello, Marco Marchetti, Andrea Cammarano, Iraj Alipourfard, Ida Ceravolo, Santo Gratteri

**Affiliations:** ^1^Section of Clinical Nutrition and Nutrigenomic, Department of Biomedicine and Prevention, University of Rome Tor Vergata, 00133 Rome, Italy; ^2^School of Applied Medical-Surgical Sciences, University of Rome Tor Vergata, 00133 Rome, Italy; ^3^Department of Experimental Medicine and Biochemical Sciences, University of Rome Tor Vergata, 00133 Rome, Italy; ^4^Department of Health Sciences, University of Magna Græcia, Viale Europa, Germaneto, 88100 Catanzaro, Italy; ^5^Department of Medical and Surgical Sciences, University of Magna Græcia, Viale Europa, Germaneto, 88100 Catanzaro, Italy; ^6^Department of Biomedicine and Prevention, University of Rome Tor Vergata, 00133 Rome, Italy; ^7^Center of Pharmaceutical Sciences, Faculty of Life Sciences, University of Vienna, A-1090 Vienna, Austria; ^8^School of Pharmacy, Faculty of Sciences, University of Rome Tor Vergata, 00133 Rome, Italy; ^9^Department of Experimental Biomedicine and Clinical Neuroscience, Ophthalmology Section, University of Palermo, Piazza Marina, 61, 90133 Palermo, Italy

## Abstract

**Introduction:**

Inflammation is associated with obesity condition and plays a pivotal role in the onset and progression of many chronic diseases. Among several nutraceutical foods, hazelnuts (Corylus avellana L.) are considered an excellent anti-inflammatory and hypolipidemic food being the second richest source of monounsaturated fatty acids among nuts and because they are rich in vitamins, minerals, and phenolic compounds.

**Materials and Methods:**

A prospective pilot clinical trial on 24 healthy volunteers who consumed daily, as a snack, 40 g of hazelnuts (261.99 kcal/1096.17 kJ) for six weeks was conducted. Anthropometric measurements, body composition analysis, and nutrigenomic analysis on 12 anti-inflammatory and antioxidant genes were evaluated at baseline (T0) and after hazelnut intervention (T1).

**Results:**

No significant changes were detected on body composition analysis after hazelnut consumption. Conversely, significant upregulation was detected for SOD1 (2^−ΔΔCt^ = 2.42), CAT (2^−ΔΔCt^ = 2.41), MIF (2^−ΔΔCt^ = 4.12), PPAR*γ* (2^−ΔΔCt^ = 5.89), VDR (2^−ΔΔCt^ = 3.61), MTHFR (2^−ΔΔCt^ = 2.40), and ACE (2^−ΔΔCt^ = 2.16) at the end of the study.

**Conclusions:**

According to emerging evidences, hazelnut consumption does not lead to weight gain probably due to the improvement of the body's antioxidant capacity by the upregulation of genes implied in oxidant reactions and inflammation.

## 1. Introduction

Inflammation is a constant feature associated with the onset and progression of many chronic degenerative diseases, dramatically increasing in western countries, and one of the leading causes of its insurgence is the high adipose tissue content [1]. White adipose tissue (WAT) is a metabolic organ able to satisfy body functional demands through the storage, in case of an extra caloric intake, or the mobilization, in case of metabolic demands, of energy. WAT is composed of adipocytes, which has marked cellular heterogeneity, vascularisation, and innervation, with a complex hormonal homeostatic system [2]. The relationship between obesity and inflammation was firstly observed in the 1990s. The metabolic dysfunction of adipocytes is the main cause of chronic inflammation in WAT, through the increasing expression of many biologically functional cytokines/chemokines considering the major mediators of inflammation in particular in obesity condition, like tumor necrosis factor-*α* (TNF-*α*), interleukin-6 (IL-6), interleukin-1*β* (IL-1*β*), interleukin-8 (IL-8), and interleukin-17D (IL-17D) [3]. The release of those inflammation cytokines determined the subsequent activation of tissue-resident macrophages [4]. In obesity, the excess of lipids and the consequent surplus of WAT can cause also an increase of reactive oxygen species (ROS) levels and the reduction of the antioxidant defenses, events that determine the induction of systemic oxidative stress [5].

Among several nutraceutical foods, tree nuts are edible dry fruits that, together with cereals and legumes, have characterized the human diet since preagricultural times, representing a high-energy and nutrient-dense food, rich in minerals, vitamins, and bioactive compounds [6]. The scientific evidence arises from both epidemiological observations and clinical trials, showing beneficial effects of nut intake on the health status. The reason why a diet rich in nuts has these health benefits is due to their high content in monounsaturated fatty acids (MUFAs) and polyunsaturated fatty acids (PUFAs) [7], sterols, and fibers and to the presence of bioactive molecules like vitamin E (*α*-tocopherol), vitamin B, arginine, and polyphenols [8, 9]. In particular, the nut antioxidant capacity was widely discussed, highlighting the bioactivity potential of nut phenolic compounds [10]. There is also a suggestion of regulatory effects of nuts on inflammation, demonstrated by the change of circulating inflammatory markers and the expression of ligands for inflammatory molecules in circulating monocytes after two weeks of an enriched nut diet [11]. Furthermore, nuts are particularly rich in calories but their enriching in diets does not change the body mass index (BMI). Actually, a higher weight loss and a greater protection against cardiovascular risk factors in subjects who consumed nuts in their diet compared to who follow other dietary plans were shown [12–14]. These observations support the theory for which the introduction of nuts in weight loss or weight control diets determines a more favourable outcome.

For all these reasons, nuts are included in the American Heart Association dietary metrics for defining ideal cardiovascular health in their recent report on setting goals for health promotion and disease reduction for 2020 [15]. Furthermore, the World Health Organization (WHO) recommend a daily intake of nuts as a fundamental part of a cardioprotective diet [16].

Among nuts, hazelnuts (Corylus avellana L.) belong to the Betulaceae family and are considered excellent anti-inflammatory and hypolipidemic food [17], being the second richest source of MUFAs among nuts and because they are rich in vitamin E and tocopherols, phytosterols (mainly *α*-sitosterol), magnesium, copper and selenium, L-arginine, polyphenols, folate, and fibers. Among the phenolic compounds, gallic acid, p-hydroxybenzoic acid, epicatechin, caffeic acid, sinapic acid, and quercetin are found in hazelnuts, with the highest unsaturated/saturated fatty acid ratio among nuts [12].

To our knowledge, only one study defined the right intake of hazelnuts to consume in order to maintain body weight [18], and few works have had a nutrigenomic approach to study the effects of hazelnut consumption [19, 20]. However, nowadays, no studies have identified the right intake of hazelnuts to be able to maintain or improve body weight and/or body composition and enhance anti-inflammatory gene expression. The mechanisms behind weight and of body composition changes given by hazelnut consumption, as well as their modulatory effect on the inflammatory genomic upstream pathway, represent a new field to discover.

The aims of this pilot study were to investigate the effect of hazelnut treatment (HNT) on body composition and on genomic response of genes related to oxidative stress and inflammation.

## 2. Materials and Methods

### 2.1. Subjects and Study Design

For this prospective clinical trial, 30 healthy volunteers were recruited and analyzed from the staff of the Clinical Nutrition and Nutrigenomic Section, Department of Biomedicine and Prevention of the University of Rome Tor Vergata. Body composition data and blood samples for genomic analysis were collected at baseline (T0) and after 6 weeks of hazelnut intervention (T1) in order to evaluate nutritional, oxidative, and inflammation statuses. In this pilot study, every recruited subject was considered a control of himself. Lifestyle habits of healthy volunteers did not change during the study period. No abnormality was presented during the study period. All participants recruited in the study authorized their participation by reading and signing the informed consent, conducted in accordance with the provisions of the Ethics Committee of Medicine, University of Rome Tor Vergata, and with the Helsinki Declaration of 1975 as revised in 1983. This protocol has been registered with Trial Registration Number ClinicalTrials.gov. ID: NCT01890070.

### 2.2. Exclusion Criteria

In order to be included in the study, subjects had to respect the following exclusion criteria: active tobacco smoking; pregnancy; breastfeeding; type 1 and type 2 diabetes; past or active cardiovascular diseases; metabolic, endocrine, liver, kidney, and autoimmune disorders; chronic viral (hepatitis C and B and HIV) and cancer diseases; corticosteroid and chronic anti-inflammatory therapy; and participation in other dietary trials.

### 2.3. Dietary Intervention

During the study period, all the recruited subjects consumed daily 40 g of hazelnuts (261.99 kcal/1096.17 kJ), cultivar Tonda Gentile Romana, provided by Coopernocciola in Vico Matrino (Viterbo, Italy). A 24 h dietary recall was performed at the baseline to all participants. All subjects were administered a standard isocaloric diet according to the following Mediterranean diet criteria: 55% of carbohydrates, 20% of proteins (>50% of vegetable derivation), <30% of lipids (on total kcal: saturated fat <10%, 6–10% polyunsaturated fatty acids (PUFA), n-6/n-3 PUFA ratio of 3 : 1, 15% of monounsaturated fatty acids (MUFA), and <1% transfatty acids), and 30 g of fiber. Caloric intake calculation of all standard isocaloric diets was based on a 24 h dietary recall evaluation for each subject. Standard diets were elaborated with a proper software (Dietosystem, DS Medica, Milan, Italy). All subjects were asked to eliminate any other type of hazelnuts or nuts from their diet.

### 2.4. Anthropometric Measurements and Body Composition Analysis

All volunteers were subjected to an anthropometric evaluation after overnight fasting. Body weight and height were measured according to previously described methods [21]. Body weight was evaluated with balance scale to the nearest 0.1 kg (Invernizzi, Rome, Italy). Height was measured with a stadiometer to the nearest 0.1 cm (Invernizzi, Rome, Italy). BMI was calculated using the following formula: BMI = body weight (kg)/height (m)^2^. Body composition analysis was assessed by Dual Energy X-Ray Absorptiometry (DXA) (i-DXA, GE Medical Systems, Milwaukee, WI, USA) [22].

### 2.5. Biochemical Analysis

Blood tests were carried out at the “Policlinico Tor Vergata (PTV)” of Rome, Italy. Analyses were performed at baseline after a 12-hour overnight fast. Blood samples (10 mL) were collected into EDTA tubes (Vacutainer®), placed in ice, and plasma was separated by centrifugation. Laboratory analysis included complete blood count, total cholesterol (TC), high-density lipoprotein cholesterol (HDL-C), low-density lipoprotein cholesterol (LDL-C), and triglycerides (Tg). Complete blood count, except serum lipid and Tg analyses, was carried out with an ADVIA®1800 Chemistry System (Siemens Healthcare). Serum lipid profile components were determined by standard enzymatic colorimetric techniques (Roche143 Modular P800, Roche Diagnostics, Indianapolis, IN, USA). Serum Tg were measured by a coupled enzymatic method on the Beckman Synchron LX20 automated system.

### 2.6. Sample Collection. RNA Extraction and Analysis

Blood samples were collected and stabilized in PAX gene Blood RNA Tubes (Pre AnalytiX, Qiagen, Hombrechtikon, Switzerland) and then stored at -80°C until use. Blood samples were pooled according to intervention times (baseline and postintervention). Total RNA was purified with a PAX gene Blood miRNA Kit, following the manufacturer's instructions (Pre AnalytiX, Qiagen, Hombrechtikon, Switzerland), and quantified through spectrophotometry (Nanodrop, Wilmington, USA). Specific RT2 Profiler PCR Arrays (Qiagen, Netherlands) were used for human oxidative stress (PAHS-065ZA, Qiagen, Netherlands) and human inflammation (PAHS-097ZA, Qiagen, Netherlands) pathways. The gene expression of the following 12 genes was analyzed: superoxide dismutase 1 (SOD1) (NCBI Accession number: NM_000454.4), catalase (CAT) (NCBI Accession number: NM_001752.3), macrophage migration inhibitory factor (MIF) (NCBI Accession number: NM_002415.1), peroxisome proliferator-activated receptor gamma (PPAR*γ*) (NCBI Accession number: NM_001354667), vitamin D receptor (VDR) (NCBI Accession number: NM_000367.2), methylenetetrahydrofolate reductase (MTHFR) (NCBI Accession number: NM_001330358.1), angiotensin I-converting enzyme (ACE) (NCBI Accession number: NM_000789.3), apolipoprotein E (APOE) (NCBI Accession number: NM_001302691.1), interleukin 6 receptor (IL6R) (NCBI Accession number: NM_000565.3), nuclear factor of kappa light polypeptide gene enhancer in B-cell 1 (NFKB1) (NCBI Accession number: NM_003998.3), insulin-like growth factor 2 receptor (IFG2R) (NCBI Accession number: NG_011785.3), and upstream transcription factor 1 (USF1) (NCBI Accession number: NM_001276373.1). Each qRT-PCR experiment was performed in triplicate and repeated at least twice, in line with the manufacturer's instructions (Qiagen, Netherlands). *β*-Actin (ACTB) (NM 001101) was used as a housekeeping gene. A comparative threshold (CT) cycle was used to determine the gene expression level. A CT value was normalized using the formula ΔCT = CT (gene) − CT (housekeeping gene). The relative gene expression levels were determined according to the following formula: ΔΔCT = ΔCT sample − ΔCT calibrator. The value used to plot relative gene expression was determined using the expression fold change (FC) = 2^–ΔΔCT^.

### 2.7. Statistical Analysis

Statistical analysis was carried out using IBM SPSS 21.0 for Windows (Armonk, NY: IBM Corp, USA). Power calculation was evaluated on total cholesterol, with a 2-sided test and an *α* = 0.05. After the Shapiro-Wilk test, a paired *t*-test or a nonparametric Wilcoxon test was performed to evaluate differences before and after hazelnut interventions. All tests were considered significant at *p* ≤ 0.05. For genomic analysis, the value used to plot relative gene expression was determined using the expression fold change (FC) = 2^−ΔΔCT^. Only genes with a FC ≥2 were considered significantly upregulated for differentially expressed genes. Conversely, genes with a FC≤0.5 were considered significantly downregulated for differentially expressed genes.

## 3. Results

### 3.1. Subjects Characteristics

**Figure 1:**
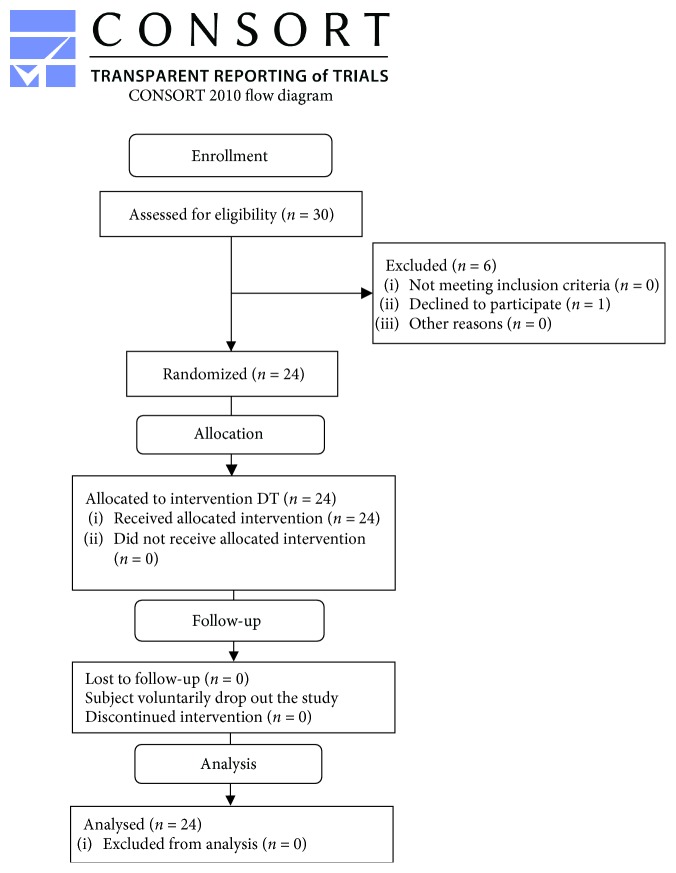
Flow chart study design.

Of the thirty subjects enrolled, one of them was excluded from the trial (subject declined to participate) (Figure 1). At the end, twenty-four subjects completed the trial. Any changes to trial outcomes after the trial commenced occurred. The power of the study was 0.61. The average age of subjects was 51.58 ± 9.37 years (58.3% male and 41.7% female) (Table 1).

### 3.2. Body Composition and Bioclinical Analysis

After 6 weeks of HNT (T1), a significant reduction of the abdominal circumference (*p* = 0.04; Δ% = −0.53%) was observed, compared to baseline (T0) values. At the same time, in DXA measurements of bone mineral content (BMC), total body lean (TBLean), android body fat (ABFat), gynoid body fat (GBFat), and total body fat (TBFat), no significant changes were detected. The same observations were highlighted for the changes in the appendicular skeletal muscle mass index (ASMMI) and *t*-score parameters (Table 2). Furthermore, among the biochemical parameters, only TC (*p* = 0.01; Δ% = −10.48%) and LDL-C (*p* = 0.01; Δ% = −12.12%) had a significant reduction after 6 weeks of HNT, as well as the TC/HDL-C ratio (*p* = 0.03; Δ% = −5.83%). No other changes were highlighted for serum lipid profile and inflammation markers (Table 2).

### 3.3. Gene Expression Data

**Figure 2:**
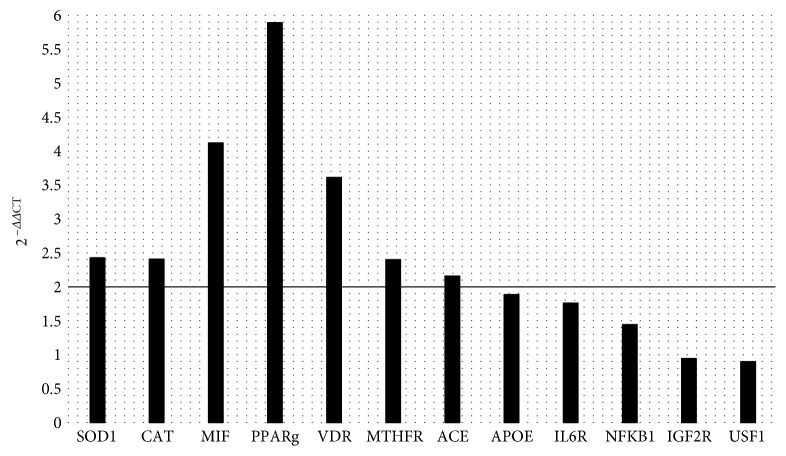
Gene expression after hazelnut treatment. Different levels of the fold change of 12 genes were analyzed: superoxide dismutase 1 (SOD1), catalase (CAT), macrophage migration inhibitory factor (MIF), peroxisome proliferator-activated receptor gamma (PPAR*γ*), vitamin D receptor (VDR), methylenetetrahydrofolate reductase (MTHFR), angiotensin I-converting enzyme (ACE), apolipoprotein E (APOE), interleukin 6 receptor (IL6R), nuclear factor of kappa light polypeptide gene enhancer in B-cell 1 (NFKB1), insulin-like growth factor 2 receptor (IFG2R), and upstream transcription factor 1 (USF1). Genes with a FC ≥ 2 were considered significantly upregulated for differentially expressed genes; genes with a FC ≤ 0.5 were considered significantly downregulated for differentially expressed genes.

Significant upregulation with a fold change exceeding the threshold set at 2 was detected for SOD1 (2^−ΔΔCt^ = 2.42), CAT (2^−ΔΔCt^ = 2.41), MIF (2^−ΔΔCt^ = 4.12), PPAR*γ* (2^−ΔΔCt^ = 5.89), VDR (2^−ΔΔCt^ = 3.61), MTHFR (2^−ΔΔCt^ = 2.40), and ACE (2^−ΔΔCt^ = 2.16) after HNT (Figure 2). No significant gene expression changes were observed for APOE, IL6R, NFKB1, IFG2R, and USF1 (0.5 < 2^−ΔΔCt^ < 2).

## 4. Discussion

Hazelnuts have been demonstrated to be able to reduce atherogenic/cardiovascular risk and inflammation [12, 18, 23]. The beneficial effects of hazelnuts on inflammation are due to the content of various nutrients and bioactive substances, and their biochemical profile depends on the cultivar and the country of origin. Nowadays, Turkey is the main country producing hazelnuts in the world, followed by Italy, where Tonda Gentile Romana is found, a typical cultivar from the Latium region, which is particularly rich in stearic, oleic, and linoleic fatty acids [24]. However, the reason why a diet rich in hazelnuts has beneficial effects on health is because of their high content of MUFAs, PUFAs, fibers, *α*-tocopherol, phytosterols, phenolic compounds, magnesium, copper, and selenium [7, 12]. Some of these components have a potential antioxidant effect through their major nonenzymatic antioxidant proprieties [25]. Constitutive enzymes function or even through their ability to increase the expression of genes involved in anti-inflammatory and antioxidant processes and/or to reduce the expression of inflammatory genes [26–28]. It is well known that the imbalance of the ROS production and the body's antioxidant capacity determines oxidative stress, which is implicated in several pathological processes. Despite the fact that 30% of obese subjects are considered metabolically healthy, a condition where insulin sensitivity, visceral fat content, and intima media thickness of the carotid artery are similar to healthy normal weight, the majority of obese patients were metabolically unhealthy, with an increased risk of cardiovascular problems, metabolic disorders, arterial hypertension, and chronic heart disease development [3]. Adipocytes represent the main cause of chronic inflammation in WAT because of the increased expression of several cytokines/chemokines and ROS production, within a reduction of the antioxidant defenses [5, 29]. In particular, obesity-related disorders are mainly due to the increasing levels of visceral adiposity tissue (VAT), which are considered extremely dangerous because they are related to the high concentration of inflammatory cytokines and obesity-related cardiometabolic problems [3]. It is well known that nuts are particularly rich in fats and calories and usually considered a hypercaloric food, but it was observed that hazelnuts or nut-enriched diets do not change weight or BMI. In fact, several epidemiological studies detected an inverse or a null association between nut consumption and BMI [17–20]. More specifically, consumption up to 60 g of hazelnuts per day did not affect weight and improve blood cholesterol levels [18].

Since 1 kg of fat contains 9000 kcal [30], an excess of 261.99 kcal per day for 6 weeks, due to the intake of 40 g of hazelnuts, would have led to a fat tissue gain of 1 kg, while no significant difference of weight and/or fat mass emerged from the results. Interestingly, as demonstrated by previous observations [12–14], although we noticed a small but significant reduction of abdominal circumference (*p* < 0.05), we did not observe any changes in weight, BMI, android, gynoid body fat, and total body fat measured by DXA after 6 weeks of a reported 1096.17 kJ/d increase in energy intake with the hazelnut-enriched diets. Furthermore, in this study, we observed a significant reduction of TC, LDL-C, and TC/HDL-C ratio according to Perna et al. [14].

There are several reasons why nut consumption does not determine weight gain and improve cholesterol profile. Firstly, nuts contain a good amount of proteins and fibers, and they have a low glycemic index, contributing to enhance satiety through the reduction of calorie intake from other food sources [31]. In addition, the high content of dietary fibers could reduce the bioavailability of cholesterol from food, and the antioxidant molecules contained in hazelnuts contribute to their antiatherogenic effect [14]. Moreover, the typical crunchy texture of nuts promotes satiety through the mechanical act of chewing, which determines the secretion of food intake regulation [32]. Secondly, previous works suggested that nut consumption leads to the energy expenditure and thermogenic effect increase, probably because of the high unsaturated/saturated fat ratio characteristic of the nuts [33]. Thirdly, lipids of nuts are not easily bioavailable [34], and then, a great part of them is excreted with feces and not accessible for metabolization [35]. Fourthly, the application of Atwater factors to nuts determined an overestimation of energy contents [36].

Another possible explanation about nut consumption and the maintenance of weight or BMI could be suggested by the antioxidant potential of hazelnuts. It is well known that some foods have peculiar antioxidant activities [37], which can affect some disturbances such as obesity. In fact, the relation between obesity and inflammation could be considered bidirectional. Few studies demonstrated that a previous inflammatory status is found before the overweight and obesity onset, becoming a possible risk factor of them [38, 39]. In the SUN study, Ramallal et al. [40] observed that subjects who follow a proinflammatory diet have a clinically relevant weight gain (3-5 kg), as compared with those in the anti-inflammatory diet group. The antioxidant capacity determined by nuts was widely discussed, highlighting the bioactivity potential of nut phenolic compounds [13].

Unfortunately, only few studies observed the effect of hazelnut consumption on inflammatory and/or anti-inflammatory gene expression, and the most relevant results were related to the upregulation of some antioxidant genes [25, 26]. In this pilot study, we confirmed that after hazelnut consumption there is an upregulation of the two of the major antioxidant enzymes, SOD1 (2^−ΔΔCt^ = 2.42) and CAT (2^−ΔΔCt^ = 2.41), which are two of the most important genes involved in the antioxidant pathway, thanks to their ability to catalyze the reaction that leads from superoxide (O_2_^−^) to oxygen and water production. Furthermore, during our trial, we observed an upregulation of MIF (2^−ΔΔCt^ = 4.12). MIF, a cytokine able to regulate innate and acquired immune responses, is usually associated to atherosclerosis progression, obesity, insulin resistance, and inflammatory diseases [41]. Recently, MIF was identified as a key regulator of antioxidant response element (ARE), a DNA enhancer that controls the expression of phase II detoxifying enzymes and cytoprotective proteins for redox homeostasis [42]. In this view, in accordance with other studies [43, 44], MIF seems to be a sensor for oxidative stress, and its upregulation, as shown in our work, could represent a new way to reduce oxidative stress, because of its role as a key regulator of ARE-mediated gene expression. Furthermore, MIF regulates the antioxidant system by binding and activating transcription factors such as Nrf2, which during oxidative stress translocate into the nucleus, inducing ARE activation. In our pilot study, hazelnut consumption is associated also with the upregulation of PPAR*γ* (2^−ΔΔCt^ = 5.89), an enzyme that cooperates in the modulation of oxidative stress, antioxidant response, and inflammatory diseases. In fact, PPAR*γ* has a reciprocal transcriptional regulation with Nrf2, acting synergically in the activation of antioxidant genes [45]. The upregulation of SOD1, CAT, MIF, and PPAR*γ* observed in this study is in line with previous works, which have demonstrated the antioxidant proprieties of a diet rich in nuts, related to the regulation of cellular pathways of atherosclerosis, inflammation, and oxidative stress [46] (Figure 2). At the same time, PPAR*γ*, which controls M2 macrophage activation, seems to be related to VDR expression. The reduction of VDR expression could determine the abolishment of PPAR*γ* effects, establishing a possible VDR-PPAR*γ* pathway that, along with vitamin D, regulates macrophage phenotype [47]. Furthermore, VDR inhibits the initiation of endothelial inflammatory diseases, like atherosclerosis, and reducing inflammatory cytokine/chemokine production in macrophages, when it is activated by vitamin D [48]. The upregulation of VDR (2^−ΔΔCt^ = 3.61) observed in our study (Figure 2) amplified the antioxidant and anti-inflammatory potential of hazelnuts. In fact, the increased VDR gene expression overlaps PPAR*γ* upregulation, suggesting a possible effect of HNT on the VDR-PPAR*γ* pathway.

High blood concentrations of homocysteine (Hcy), an important intermediary in the metabolism of methionine and cysteine, are another potential inflammatory factor. Hcy induces the production of several proinflammatory cytokines, and at the same time, in endothelial cells, high levels of Hcy induce the oxidative inactivation of nitric oxide with the collaboration of several ROS. Furthermore, Hcy plays an important role in the conservation of intracellular glutathione pools and then in oxidative stress conditions. A full MTHFR expression and activity are strictly necessary for the proper recycle of Hcy. Its reduced gene expression could lead to mental disorders, developmental delays, cardiovascular diseases, and cancer [49]. The increased MTHFR gene expression (2^−ΔΔCt^ = 2.40) observed in this pilot study after HNT could reinforce the antioxidant effect of hazelnut consumption (Figure 2). In fact, if MTHFR gene is upregulated, we can speculate a reduction of ROS production and inflammation.

Ordinary nut consumption, maybe due to the polyphenol content, is associated with beneficial effects on blood pressure [47]. High blood pressure is one of the risk factors associated with oxidative stress and inflammation. Usually, increased levels of vascular ACE, the key regulator of the renin-angiotensin system and kallikrein-kinin system, are associated with increased blood pressure [50]; however, evidences have been reported between ACE gene expression and hypertensive condition [51]. In our study, hazelnut consumption leads to an increase of ACE gene expression (2^−ΔΔCt^ = 2.16) (Figure 2), but not to a significant change in SBP or DBP (*p* > 0.05), strengthening the observation of Mohammadifard N. et al. that maybe only mixed nut and pistachio consumption could be associated with better blood pressure outcomes [52].

## 5. Conclusions

In conclusion, the reasons why nuts do not determine weight gain are various, like satiety induction, energy expenditure, thermogenesis increase, and the low nutrient bioavailability. According to emerging evidences, hazelnut antioxidant capacity could be another reason. Although this pilot study was not without limitations, as the lack of biochemical analysis, serum protein levels of investigated genes, antioxidant markers, and the limited sample size, our results suggest that an ordinary consumption of small amount of hazelnuts could be a nutritional treatment for all chronic degenerative diseases underlying oxidative stress and inflammation reinforcing the WHO recommendation. Furthermore, the lack of weight gain and, more importantly, the lack of fat mass gain with the hazelnut consumption, which is still considered a high-calorie food, could represent an encouragement for the inclusion of this food not only in the anti-inflammatory dietary patterns but also in all weight loss diets. Our data should be confirmed on a larger number of subjects, with a prospective long-term controlled trial.

## Figures and Tables

**Table 1:** Anthropometric and clinical baseline characteristic of study subjects.

Parameter (*n* = 24)	Male			Female		
Frequency (*n*, %)	(*n* = 14; 53.8%)			(*n* = 10; 41.7%)		
	Median	P25	P75	Median	P25	P75
Age (years)	56.50	52.00	60.50	47.50	41.25	56.25
SBP (mmHg)	125.50	112.25	140.00	108.00	101.75	121.50
DBP (mmHg)	74.50	65.00	88.25	72.50	63.75	75.50
Height (cm)	172.00	165.00	173.25	161.00	155.75	162.00
Weight (kg)	75.00	69.50	82.95	63.95	58.83	78.68
BMI (kg/m^2^)	26.23	24.34	27.36	25.37	22.99	30.07
Neck circumference (cm)	41.00	39.75	42.63	37.00	35.38	38.25
Waist circumference (cm)	88.50	82.75	92.25	81.50	72.00	85.88
Abdominal circumference (cm)	94.00	87.13	97.00	92.75	86.75	99.75
Hip circumference (cm)	97.50	94.00	99.25	100.50	96.00	114.13
Waist/hip ratio	0.91	0.87	0.96	0.79	0.73	0.84
TBFat (kg)	19.30	16.20	22.60	22.90	20.51	37.41
TBFat (%)	25.60	22.28	28.58	38.45	33.05	42.28
ABFat (%)	33.15	25.08	37.53	43.00	33.98	51.75
GBFat (%)	25.70	22.98	27.15	39.35	36.88	49.63
TBLean (kg)	53.94	48.04	58.36	38.39	36.44	40.93
BMC (g)	2896.00	2631.75	3108.50	2313.00	2059.50	2634.75
ASMMI	8.74	8.37	9.29	6.67	6.42	7.59
*t*-score	-0.40	-0.93	0.13	0.25	-1.13	1.60
Neutrophils (K/*μ*L)	2850.00	2000.00	3530.00	3070.00	2190.00	3360.00
Lymphocytes (K/*μ*L)	1800.00	1240.00	2000.00	2120.00	1160.00	2540.00
Platelets (K/*μ*L)	191000.00	167000.00	204000.00	243000.00	173000.00	256000.00
TC (mg/dL)	193.00	149.00	245.00	214.00	159.00	270.00
HDL-C (mg/dL)	50.00	43.00	66.00	67.00	38.00	70.00
LDL-C (mg/dL)	131.00	80.00	159.00	146.00	75.00	178.00
Tg (mg/dL)	95.00	42.00	113.00	99.00	54.00	124.00
NLR	1.46	1.11	2.40	1.52	0.83	2.22
PLR	120.00	77.78	129.41	106.60	65.04	186.72
Tg/HDL-C	1.44	0.68	2.51	1.81	0.77	2.24
TC/HDL-C	3.86	2.40	4.00	3.87	2.34	4.07
kcal/diet	1960.00	1650.00	2050.00	1840.00	1230.00	2050.00

Anthropometric and clinical characteristic at baseline (T0) and after HNT (T1). Results are expressed as the median, minimum, and maximum for each parameter. A paired *t*-test (a) or a nonparametric Wilcoxon test (b) was performed to evaluate differences before and after hazelnut intervention. All tests were considered significant at *p* ≤ 0.05. SBP: systolic blood pressure; DBP: diastolic blood pressure; TBFat: total body fat; ABFat: android body fat; GBFat: gynoid body fat; TBLean: total body lean; BMC: bone mineral content; ASMMI: appendicular skeletal muscle mass index; TC: total cholesterol; HDL-C: high-density lipoprotein cholesterol; LDL-C: low-density lipoprotein cholesterol; Tg: triglycerides; NLR: neutrophil-lymphocyte ratio; PLR: platelet-lymphocyte ratio.

**Table 2:** Anthropometric and clinical characteristic at baseline (T0) and after hazelnut treatment (HNT) (T1).

	Baseline (T0)		HNT (T1)		*Δ*%	*p*
	Median	Min–Max	Median	Min–Max		
SBP (mmHg)	116.50	96.00-169.00	120.00	100.00-150.00	3.00	0.81^a^
DBP (mmHg)	73.00	57.00-93.00	75.00	60.00-86.00	2.74	0.28^a^
Weight (kg)	71.40	53.50-93.00	71.05	53.50-93.00	-0.49	0.46^a^
BMI (kg/m^2^)	25.95	20.64-35.60	25.76	20.64-35.44	-0.71	0.55^a^
Neck circumference (cm)	39.50	33.00-44.00	40.00	34.00-43.00	1.27	0.71^b^
Waist circumference (cm)	86.25	66.50-101.00	85.00	66.00-103.00	-1.45	0.90^a^
Abdominal circumference (cm)	94.00	73.00-110.00	93.50	81.00-110.50	-0.53	0.04^a^
Hip circumference (cm)	98.25	92.00-117.00	99.00	91.00-116.00	0.76	0.44^b^
Waist/hip ratio	0.87	0.71-1.03	0.87	0.69-1.00	0.00	0.34^a^
TBFat (kg)	34.75	14.60-53.10	34.95	13.50-52.60	0.58	0.89^b^
TBFat (%)	29.65	16.3-54	29.05	18.00-53.30	-2.02	0.73^a^
ABFat (%)	28.75	16.10-48.30	28.80	15.40-48.00	0.17	0.15^a^
GBFat (%)	21.08	12.02-38.02	21.34	12.49-38.13	1.21	0.89^a^
TBLean (kg)	47.63	32.87-70.81	48.09	32.40-68.42	0.97	0.34^a^
BMC (g)	2703.00	1667.00-3742.00	2622.00	1692.00-3627.00	-3.00	0.29^a^
ASMMI	8.37	5.98-10.28	8.05	6.06-10.57	-3.83	0.11^a^
*t*-score	-0.1000	-1.90-1.90	-0.1000	-1.90-1.90	0.00	0.87^a^
Neutrophils (K/*μ*L)	2970.00	2220-6860	3245	2170-8481	11.71	0.40^a^
Lymphocytes (K/*μ*L)	1695.00	1160.00-2680.00	1660.00	754.00-2700.00	-7.11	0.18^b^
Platelets (K/*μ*L)	194500.00	166000.00-293000.00	214000.00	148000.00-324000.00	6.01	0.14^a^
TC (mg/dL)	181.00	149.00-214.00	167.00	102.00-200.00	-10.48	0.01^a^
HDL-C (mg/dL)	51.50	35.00-79.00	47.50	37.00-80.00	-6.93	0.22^a^
LDL-C (mg/dL)	114.00	75.00-146.00	103.00	47.00-125.00	-12.12	0.01^a^
Tg (mg/dL)	100.50	42.00-170.00	82.50	44.00-151.00	-11.99	0.20^a^
NLR	2.08	0.83-4.48	2.14	0.95-6.68	28.87	0.18^b^
PLR	127.08	61.94-198.28	135.69	64.91-309.02	18.10	0.08^a^
Tg/HDL-C	2.02	0.68-3.55	1.87	0.61-3.02	-5.61	0.55^b^
TC/HDL-C	3.71	2.28-5.03	3.27	2.23-4.6	-5.83	0.03^a^
kcal/diet	1965.00	1230.00-2780.00	2226.99	1491.99-3041.99	11.38	0.03^a^

Anthropometric and clinical characteristic at baseline (T0) and after HNT (T1). Results are expressed as the median, minimum, and maximum for each parameter. A paired *t*-test (a) or a nonparametric Wilcoxon test (b) was performed to evaluate differences before and after hazelnut intervention. All tests were considered significant at *p* ≤ 0.05. SBP: systolic blood pressure; DBP: diastolic blood pressure; TBFat: total body fat; ABFat: android body fat; GBFat: gynoid body fat; TBLean: total body lean; BMC: bone mineral content; ASMMI: appendicular skeletal muscle mass index; TC: total cholesterol; HDL-C: high-density lipoprotein cholesterol; LDL-C: low-density lipoprotein cholesterol; Tg: triglycerides; NLR: neutrophil-lymphocyte ratio; PLR: platelet-lymphocyte ratio.

## Data Availability

The data used to support the findings of this study are available from the corresponding author upon request.

## Abbreviations

ABFat: Android body fat
ACE: Angiotensin I-converting enzyme
ARE: Antioxidant response element
APOE: Apolipoprotein E
ASMMI: Appendicular skeletal muscle mass index
BMI: Body mass index
BMC: Bone mineral content
CAT: Catalase
CCL5: Copper chaperone for superoxide dismutase
DBP: Diastolic blood pressure
DXA: Dual energy X-ray absorptiometry
FC: Fold change
GBFat: Gynoid body fat
GPX1, GPX2: Glutathione peroxidases 1 and 2
GSR: Glutathione reductase
HNT: Hazelnut treatment
HSPA1A: Heat shock protein 1A
Hcy: Homocysteine
IFG2R: Insulin-like growth factor 2 receptor
IL-17D: Interleukin-17D
IL-1*β*: Interleukin-1*β*
IL-6: Interleukin-6
IL-8: Interleukin-8
IL6R: Interleukin 6 receptor
KRT1: Keratin
MIF: Macrophage migration inhibitory factor
MBL: Mannose-binding lectin (protein C) 2 soluble
MTHFR: Methylenetetrahydrofolate reductase
MUFAs: Monounsaturated fatty acids
NFKB1: Nuclear factor of kappa light polypeptide gene enhancer in B-cell 1
PPAR*γ*: Peroxisome proliferator-activated receptor gamma
PUFAs: Polyunsaturated fatty acids
ROS: Reactive oxygen species
SOD1: Superoxide dismutase 1
SBP: Systolic blood pressure
TXNRD1: Thioredoxin reductase 1
TBFat: Total body fat
TBLean: Total body lean
TNF-*α*: Tumor necrosis factor-*α*
USF1: Upstream transcription factor 1
VAT: Visceral adiposity tissue
VDR: Vitamin D receptor
WAT: White adipose tissue
WHO: World Health Organization.

## Supplementary Materials

Supplementary Materials Graphical Abstract: effects of 6 weeks of hazelnut administration on body composition and gene expression. Effects of hazelnut administration on body composition and gene expression of 7 genes belonging to the oxidative stress and the related involved pathway. Superoxide dismutase 1 (SOD1), catalase (CAT), macrophage migration inhibitory factor (MIF), peroxisome proliferator-activated receptor gamma (PPAR*γ*), vitamin D receptor (VDR), methylenetetrahydrofolate reductase (MTHFR), angiotensin I-converting enzyme (ACE), interleukin-10 (IL10), RAS, M1 and M2 macrophages, nuclear factor- (erythroid-derived 2) like 2 (Nrf2), and antioxidant response element (ARE).
