# Supplementary figures and images for: A Hazelnut-Enriched Diet Modulates Oxidative Stress and Inflammation Gene Expression without Weight Gain
Source: Oxid Med Cell Longev. 2019 Jul 4;2019:4683723. doi: 10.1155/2019/4683723 (PMC6637671; doi:10.1155/2019/4683723)

Graphical Abstract

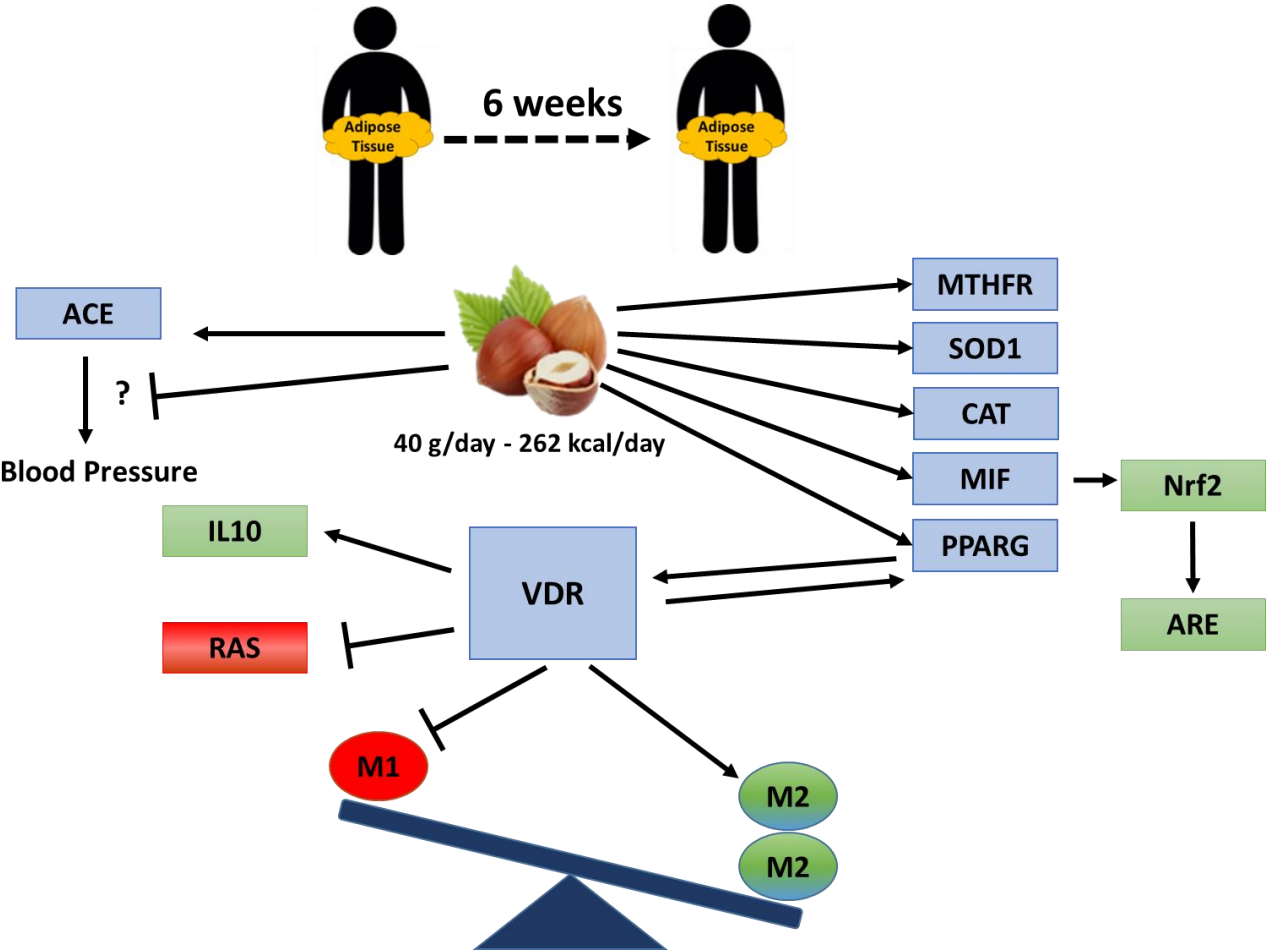

Supplement: Supplementary Materials — Graphical Abstract: effects of 6 weeks of hazelnut administration on body composition and gene expression. Effects of hazelnut administration on body composition and gene expression of 7 genes belonging to the oxidative stress and the related involved pathway. Superoxide dismutase 1 (SOD1), catalase (CAT), macrophage migration inhibitory factor (MIF), peroxisome proliferator-activated receptor gamma (PPARγ), vitamin D receptor (VDR), methylenetetrahydrofolate reductase (MTHFR), angiotensin I-converting enzyme (ACE), interleukin-10 (IL10), RAS, M1 and M2 macrophages, nuclear factor- (erythroid-derived 2) like 2 (Nrf2), and antioxidant response element (ARE). [file 4683723.f1.pdf]
